# Implementation and Monitoring of a Gestational Trophoblastic Disease Management Program in a Tertiary Hospital in Morocco: Opportunities and Challenges

**DOI:** 10.1155/2017/5093472

**Published:** 2017-04-16

**Authors:** Imane Khachani, Mohamed Hassan Alami, Rachid Bezad

**Affiliations:** National Center for Reproductive Health, University Hospital Ibn Sina, University Mohammed V Rabat, No. 1, Rue Soekarno, Rabat, Morocco

## Abstract

*Objective.* Gestational Trophoblastic Disease (GTD) management requires clear guidelines for diagnosis, treatment, and follow-up. Unequal management skills among practitioners, inadequate treatment, irregular surveillance, and drop-out are common in resource-limited settings and can lead to life-threatening complications and morbidities. To address these challenges, we implemented a GTD Management Program at the National Center for Reproductive Health in Rabat, Morocco.* Methods and Program Description.* In-depth review of management protocols was carried out, and concise guidelines were developed, with targeted training for physicians. A physical space and a weekly fixed GTD consultation were set, and personalized follow-up was established for each patient. An electronic database documenting patients' surveillance was created, allowing immediate outreach in case of irregularities.* Results.* During the period from October 2013 to June 2016, 50 patients were included in this program. Patients' mean age was 33 years; 92% were illiterate and 82% had a low socioeconomic status. 68% had a positive evolution, while 32% developed gestational trophoblastic neoplasia, requiring 2 to 6 chemotherapy sessions. An average of 2.8 outreach reminders were necessary for each patient. 94% fully adhered to the program of care and completed properly their follow-up.* Conclusion.* Implementation and thorough monitoring of this program helped optimize patients' care, avoiding drop-outs and delays in diagnosing and treating complications.

## 1. Introduction

Gestational Trophoblastic Diseases are a heterogeneous group of entities defined by the abnormal growth of trophoblast cells inside the uterus after conception, with different clinical presentations, imaging features, histological characteristics, and therapeutic options [1]. Their prognosis is generally good but relies on accurate diagnosis, adequate treatment, and thorough posttreatment surveillance for early diagnosis of complications.

Many studies examining GTD patients' therapeutic outcomes and quality of care over the past three decades have highlighted the frequent absence of clear guidelines and protocols for diagnosis and treatment in many healthcare settings, leading to inadequate risk classification, inaccurate treatment, and insufficient posttherapeutic surveillance or even drop-out [2, 3]. To tackle these shortfalls, GTD reference centers and observatories were established and evaluations have shown a clear improvement in patients' care and disease prognosis [4, 5]. In Morocco, little research has been conducted on GTD and the few studies published raised alarming concerns regarding late diagnosis, irregular surveillance, frequent drop-out, and common delayed diagnosis of preventable complications [6, 7]. An evaluation of GTD management was carried out at our National Center for Reproductive Health, one of the two public tertiary referral hospitals of the region of* Rabat-Salé-Zemmour-Zaërs*, and revealed the absence of standardized practices in diagnosis, treatment, and patients' surveillance among practitioners; scarce knowledge of patients on their condition due to insufficient explanations; irregular follow-up; frequent drop-out; and absence of tools and support materials to document patients' management. This motivated the implementation of a GTD management program, in order to optimize patients' care.

## 2. Methods and Program Description

This prospective study was conducted by descriptive and analytical method from October 2013 to June 2016 and aimed at assessing the implementation of a GTD management program at the National Center for Reproductive Health in Rabat.

The implementation process included the following:

- Development of standardized protocols for the diagnosis, treatment, and surveillance of GTD, based on the recommendations of the* International Federation of Gynecology and Obstetrics (FIGO)*, the* French College of Gynecologists and Obstetricians (CNGOF),* and the* American College of Obstetrics and Gynaecology (ACOG)* [1, 8, 9].
- These protocols established the following:
  - GTD clinical, biological, and imaging criteria with a standardized complete check-up at admission for both patients consulting for the first time and those referred from other structures with clinical and/or biological and/or imaging suspicion of GTD, including for the latter a new ultrasound scan and baseline quantitative hCG plasma assay at the Center's laboratory, in order to establish a first assessment and surveillance according to the Center's guidelines and harmonize patients' management.
  - * Therapeutic management options*, to be approved for each patient at the daily medical staff meeting.
  - * Systematic ultrasound-guided vacuum aspiration* to reduce second aspirations for intrauterine trophoblastic retentions as recommended by the FIGO [9].
  - * Mandatory histopathology examination* for each suspected GTD vacuum aspiration product.
  - * Systematic postvacuum aspiration consultation 10 days after the procedure*, including a clinical examination, ultrasound scan to assess uterine vacuity, and control quantitative hCG plasma assay. Intrauterine retention was defined at sonography by the persistence of an image inside the uterine cavity with an anteroposterior axis above 17 mm. Retention diagnosis was followed by readmission and second aspiration.
  - * Centralized quantitative plasma hCG tests*, performed at the Center's laboratory for all patients. Immunometric hCG assays were used and results were expressed in mUI/mL. Levels below 5 mUI/mL were considered “negative.” Monitoring intervals for surveillance were fixed by consensus each 10 days until 3 successive “negative” values during the first 6 months, followed by a monthly test during one year. Intervals of 10 days instead of the weekly recommended ones had the objective of reducing surveillance burden—in terms of both laboratory tests cost and patients' visits to the hospital—without compromising quality of care. Given the absence of referent histopathologist in GTD in Morocco, this surveillance scheme was adopted for both complete hydatidiform moles (CHM) and partial hydatidiform moles (PHM) to ensure higher security for patients and avoid the consequences of a potential underestimated diagnosis.
  - Gestational Trophoblastic Tumors (GTTs) diagnosis criteria and therapeutic approaches, based on the FIGO TTG staging and recommended chemotherapy protocols [10, 11].
- Design of an Information, Education, and Communication (IEC) module, including
  - detailed and simplified explanations of the characteristics of GTD, developed in* Darija Arabic*, the local dialect;
  - awareness-raising messaging on the importance and benefits of regular surveillance and risks of irregular monitoring or drop-outs;
  - systematic contraceptive counselling, in collaboration with the Department of Family Planning of the Center.
- * Creation of GTD standardized clinical files* to uniformly document patients' history, therapeutic management, and surveillance outcomes. Each file included a semilogarithmic graph to record hCG measurements and better visualize patients' evolution.
- * Creation of an on-site GTD Excel Electronic Registry*, including all patients' records, to be filled out and updated by the practitioner in charge of the weekly consultation. A section of the Registry indicated the date of the next scheduled consultation and hCG test for each patient, allowing active outreach (phone call or text message) in case of no show.
- * Equipment of a physical space* to set the weekly fixed consultation (every Thursday).
- * Centralization of paraclinical examinations*: ensuring convenient on-site management of patients, quick access to results for practitioners, and free access to all patients under Ministry of Health supported National Medical Assistance Regimen [12].

## 3. Results

From October 2013 to June 2016, 21.701 patients were admitted for delivery at the National Center for Reproductive Health. 50 patients were included in the GTD management program, which corresponds to an incidence of 2.3 for 1000 deliveries. This cohort comprehended all patients consulting directly or referred to the Center with GTD suspicion, for whom histopathological evidence of the disease was established during their management and treatment at the Center.

### 3.1. Epidemiological Data

**Figure 1:**
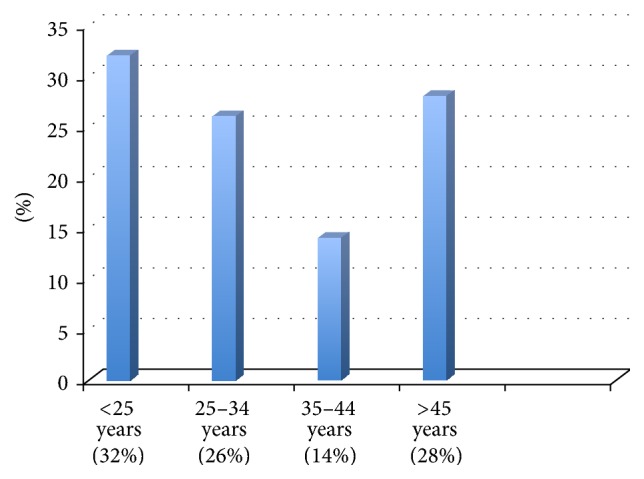
Distribution of patients according to age groups.

Mean age of patients was 33 years, with extremes ranging from 19 to 51 years; and 32% of our cohort was under 25 (Figure 1).

Regarding parity, 42% were nulliparous; 40% parity from 2 to 4, and 18% multiparous.

**Figure 2:**
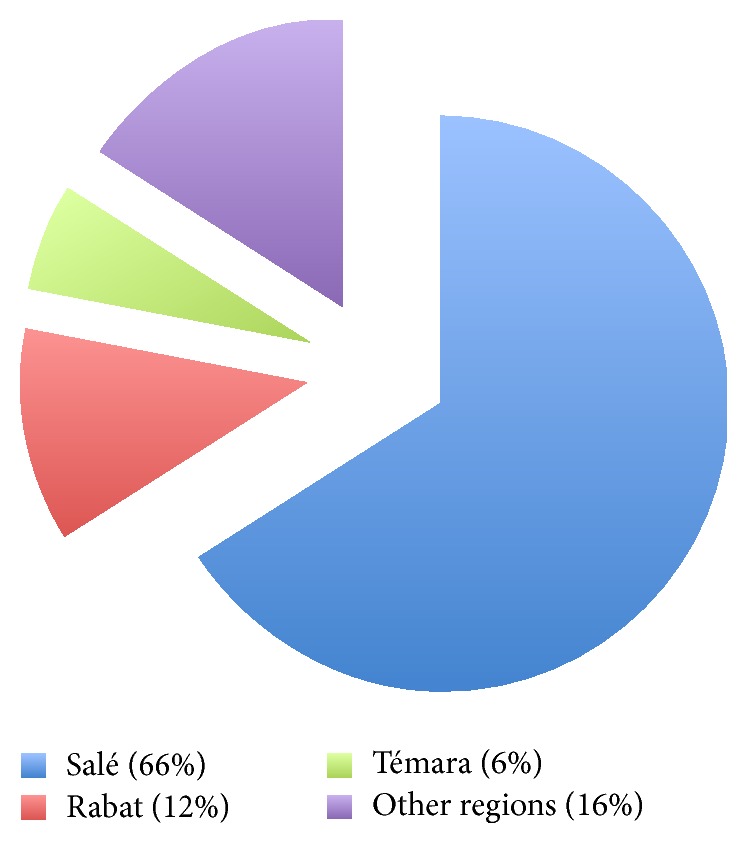
Geographic origin of patients.

84% of patients were from* Rabat-Salé-Zemmour-Zaërs* region, which is covered by the Center as a tertiary referral structure. 66% were from the city of Salé, followed far behind by the cities of Rabat (12%) and Témara (6%). The remaining 16% were from the regions of* Tanger-Tétouan-Al Hoceima* (8%);* Gharb-Cherarda-Beni Hsan* (6%); and* Meknes-Tafilalet* (2%) (Figure 2). Our Center was not their regional referral institution but they chose it for the reasons reported below:

- Trust for the quality of care (14%)
- Partner working in Rabat (2%)

The large majority of patients were illiterate (92%) and had a low socioeconomic status (82%). The second group of patients were all beneficiaries of the National Medical Assistance Regimen, which allowed free access to consultations and paraclinical tests in the Center.

### 3.2. GTD Management

The leading cause of consultation for 97% of patients was vaginal bleeding.

**Figure 3:**
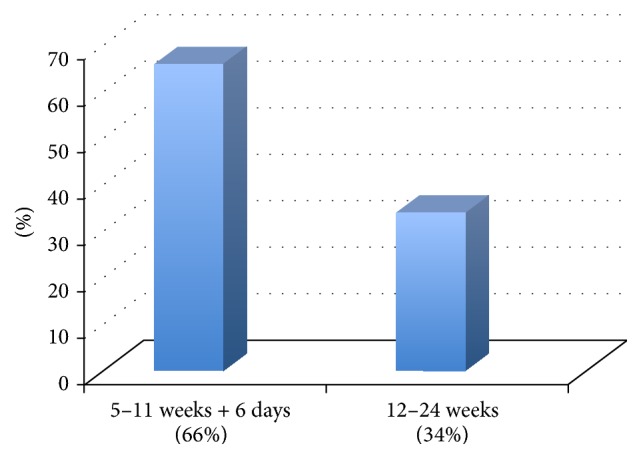
Gestational age at diagnosis of GTD.

Mean gestational age (GA) was 12 weeks with extremes ranging from 5 to 24 weeks. 66% consulted between 5 and 11 weeks + 6 days and 34% between 12 and 24 weeks (Figure 3).

**Figure 4:**
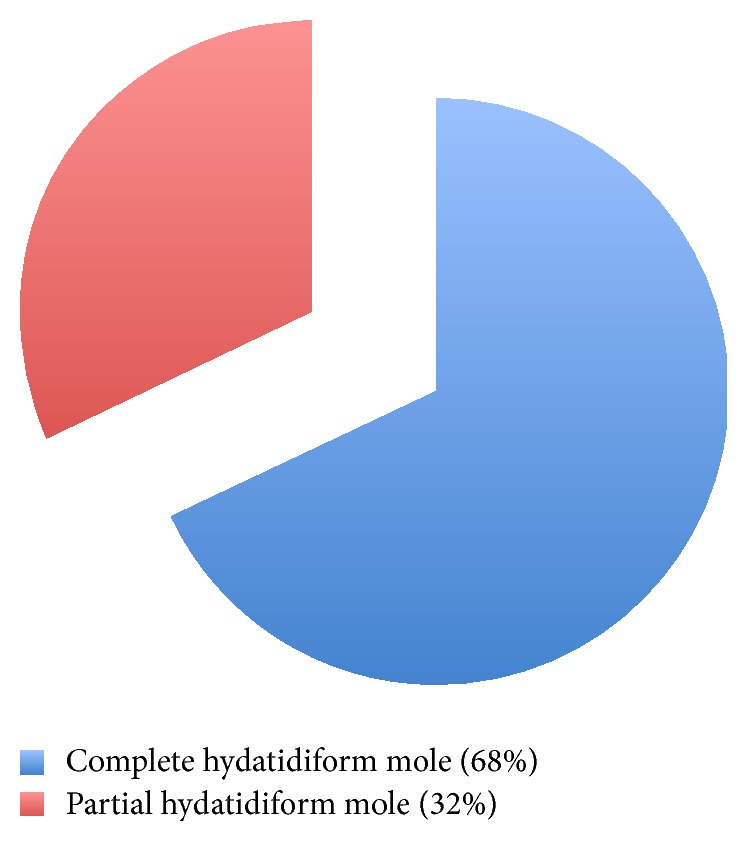
Histopathological analysis outcomes.

All vacuum aspirations were performed at the Center and all aspiration products sent for histopathological analysis. It revealed 34 cases of CHM (68%) and 16 PHM (32%) (Figure 4).

26% of patients had intrauterine retention at their control ultrasound scan and were admitted for a second aspiration.

### 3.3. Follow-Up and Evolution

**Figure 5:**
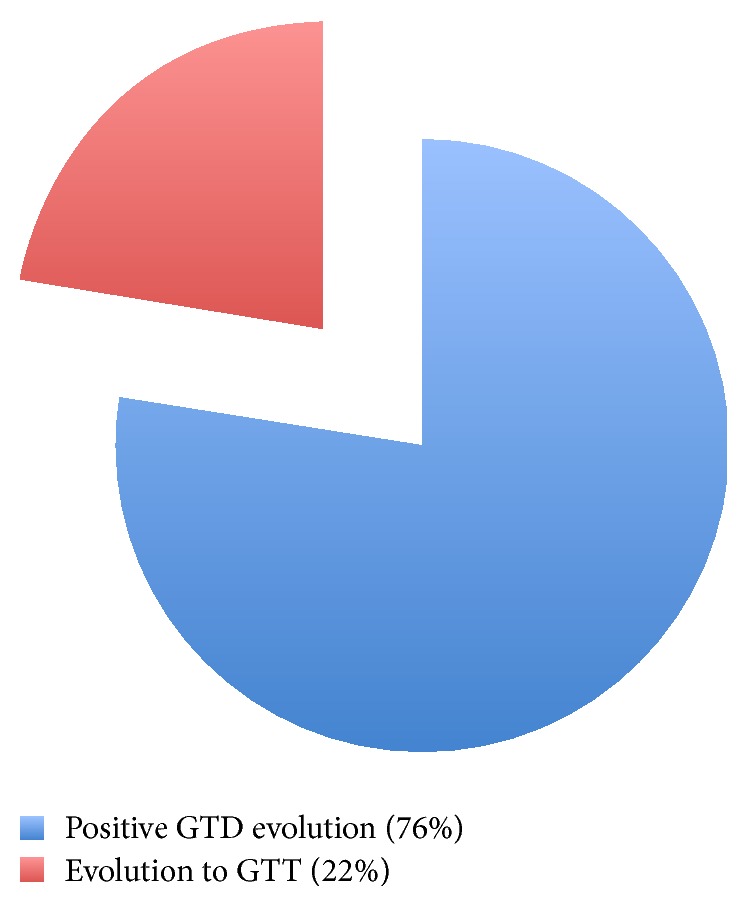
Patients' evolution.

36 patients (72%) had a positive evolution, with plasma hCG levels decreasing and reaching below 5 mUI/mL threshold between 8 and 24 weeks after vacuum aspiration. 11 of them completed the surveillance protocol and were declared cured. 11 patients (22%) presented a TTG (Figure 5).

Patients' epidemiological and management data were synthesized in Table 1.

*For the patients with TTG (Table 2),*

- gestational age at initial GTD diagnosis was before 11 weeks + 6 days for 3 of them (27%) and beyond 12 weeks for 8 (73%);
- all initial histopathological examinations had revealed a complete hydatidiform mole;
- 10 were staged low risk and one was high risk: the 10 low risk patients had their chemotherapy at the Center: a total of 2 to 6 sessions before drop of hCG plasma level below 5 mUI/mL. The high risk patient was referred to the National Oncology Institute for management.

### 3.4. Adherence

94% of patients adhered to the surveillance protocol. One patient (from the* Meknes-Tafilalet* region) dropped out after the first postvacuum aspiration control consultation. She could not be reached through the contact details she left at her admission. Two patients got pregnant before completing their surveillance protocol, respectively, at 12 and 20 weeks after vacuum aspiration. Both had reached an hCG plasma level under the 5 mUI/mL threshold before becoming pregnant. Their pregnancy was closely monitored at the Pregnancies with High Risk Department of the Center and was carried to term with no complications.

An average of 2.8 outreach reminders (phone calls and/or text messages) per patient were necessary to ensure continued adherence to the surveillance protocol. Extremes ranged from 1 to 6 phone calls or text messages to remind patient with a missed appointment and get the patient to consult at the hospital.

## 4. Discussion

The results of this study have demonstrated the multiple benefits of implementing a GTD management program in a tertiary hospital in a low-resource setting.

### 4.1. Contributing to Document GTD Local Epidemiology

The program led to the creation of a comprehensive database at the Center, documenting patients' epidemiological characteristics and highlighting some potential GTD risk factors in the local context.

The incidence of GTD in our Center was 2.3/1000 deliveries, close to the one reported by Khabouze et al. in a study conducted in the other University Maternity Hospital of Rabat between 1990 and 1997 (2.1/1000 deliveries) [6]. These similar numbers may reflect the global incidence in the* Rabat-Salé-Zemmour-Zaërs* region, where the large majority of our patients came from (84%), although as argued by Khabouze et al. they should be interpreted with caution [6]. Indeed, since most spontaneous miscarriages and vacuum aspiration products for pregnancy loss are not systematically submitted for histopathological analysis due to cost constraints, the real incidence of GTD in the region remains difficult to establish, and the abovementioned numbers are probably underestimates. In the rest of Morocco, little data is available on GTD. In the neighboring Great Casablanca region, Boufettal et al. reported an incidence of 4.2 cases per 1000 deliveries at the Ibn Rochd University Hospital between 2000 and 2009, close to twofold our incidence [7]. Bearing in mind Khabouze argument, making any interpretation or comparison difficult, interhospital incidence variations could probably be attributed to differences in patient's fluxes and referral management systems adopted by each regional center.

In the Middle East and North Africa region, data on GTD incidence is also scarce and available statistics from local studies show a great variability. Oum reported 6.6 GTDs/1000 deliveries at Al Azhar hospital in Egypt [13], while, in Turkey, the study of Çakmak et al. revealed an incidence of 1.2/1000 deliveries in the province of Tokat [14]. Similarly to Morocco, the absence of nation-wide data due to the lack of unified reporting mechanisms and absence of a structure centralizing these data makes it challenging to document the real incidence of the disease.

While considering the epidemiological characteristics of our patients in more detail, our study showed that the age group under 25 was particularly affected in our context, accounting for the third of the cohort, along with nulliparous patients who formed nearly half of it. This pattern was reported by Khabouze et al. and Boufettal et al. in Morocco [6, 7] but also worldwide, as published in the research of Parazzini et al. in Italy; Abboud et al. in France; Kuyumcuoglu et al. in Turkey; Karimi-Zarchi et al. in Iran; and Mourali et al. in Tunisia [15–19], and confirms young age and nulliparity as major risk factors of the disease. These studies also found a low socioeconomic status for most affected patients. This is particularly striking in the Moroccan context, where a low socioeconomic status was reported for over 80% of patients, including those in our study [6, 7]. Analysis of our patients' geographic origin found a large majority from* Salé*, a city often described as a “poverty pocket” in the region of* Rabat-Salé-Zemmour-Zaërs*, with over 20% of its population living under the poverty line, further confirming the low socioeconomic status pattern of GTD patients. In the nineties, Flam et al. defined low socioeconomic status as a major GTD risk factor in Sweden and the publications of Smith et al. in New Mexico over 25 years confirmed a higher incidence among young Hispanic migrant workers, mostly in precarious situation [20, 21]. Some authors attributed this pattern to poor protein diet; others mentioned the potential role of Vitamin A or Folic Acid deficiency [22, 23]. It seems that there are multiple environmental factors involved in increased GTD risk, but further research is necessary to affirm their exact role in generating or favoring the genesis of GTD. In addition, our study also explored their literacy level—directly related to socioeconomic status in the Moroccan context—and found that the large majority (92%) had never been to school and did not know how to read or write. This was initially considered as a major challenge for setting an effective GTD management program and ensuring correct adherence to the necessary surveillance. However, the design of an IEC module, using the local dialect and an accessible, culturally sensitive awareness-raising messaging, was likely instrumental in enhancing adherence to the program and fostering an effective patient-practitioner therapeutic partnership. This observation supports the necessity to include a patient-specific approach to GTD management programs in similar contexts, taking into account the characteristics of the population targeted and their specific IEC needs.

Regarding patients' admission, the large majority of our cohort consulted for vaginal bleeding (97%) and were diagnosed at a mean GA of 12 weeks, that is to say, mostly at the end of the first trimester of pregnancy. It is also interesting to note that nearly one patient out of 3 (34%) was diagnosed during the second trimester, which is considerably late. While similar data were found in studies in other low and middle income countries, Egypt, Turkey, Iran, and Tunisia [13, 14, 18, 19], in France, Abboud et al. reported only 55% of GTDs diagnosed after the patient consulted for vaginal bleeding. The remaining 45% were discovered at routine first-trimester ultrasound scan between 8 and 10 weeks, prior to onset of symptoms. Mean GA at discovery of GTD in their series was also earlier, at 9 weeks [16]. These findings highlight the importance of systematic first-trimester ultrasound examination, as it allows early diagnosis and management of the disease, key elements for a better prognosis. First-trimester ultrasound scan is not systematic in Morocco, and it remains rarely performed in the public health sector in absence of clinical warning signs. This is mainly due to limited trained human resources and scarce availability of sonographs and partly explains the delayed gestational age at diagnosis of the disease. The findings of our study suggest the need to investigate possibilities of implementing routine first-trimester ultrasound scan, at least for populations at risk, which could be determined according to our local context and capacities.

### 4.2. Structuring and Improving GTD Management in Our Center

The program enabled enhanced organization and management of patients, with a better understanding of the multiple constraints affecting patients' care and quality of care.

All 50 cases were thoroughly documented and their management was approved according to the adopted guidelines. All vacuum aspirations were performed respecting the norms of security established in our protocol and recommended by the ACOG and FIGO [1, 9]. No incidents or complications were reported. 26% of patients had a trophoblastic retention image at control ultrasound scan and underwent a second aspiration, as recommended by the CNGOF [8]. Retention after first evacuation is seldom documented in the literature. The study of Abboud et al. did not report any second aspiration needed for the 9 patients of their series. In the “First Epidemiological Data from the French Trophoblastic Disease Reference Center” study—published by the first French Center of Excellence in GTD Management in Lyon—retention rate after evacuation and need for a second aspiration was 25%, similar to ours [24]. Retention is likely related to the initial size of the mass but also to the skills of the practitioner performing the procedure [9, 24]. This explains the FIGO's recommendation of proceeding with the evacuation by a senior trained practitioner when uterine size is above 16 weeks' size [9].

Regarding histopathological analysis, the products examined were complete hydatidiform moles for 68% and partial moles for 32%. This roughly “two-thirds/one-third” distribution with a large majority of complete forms was described by the ACOG and CNGOF [1, 8] and was similarly found in the studies of Abboud et al. [77%/23%]; Sharifi et al. [76%/24%]; and Almasi et al. [84%/16%] [16, 25, 26]. Despite these similarities, the interpretation of our findings should be cautious since, in absence of a referent histopathologist with specific training in GTD diagnosis, strict differentiation between both could be uncertain.

In terms of patients' evolution, nearly three-quarters (72%) were declared cured after fully completing their one-year surveillance protocol with no relapse or complication, while a quarter (22%) presented GTT diagnosed during their follow-up. Positive evolution of GTD under surveillance varies greatly depending on studies between 50% and 90% and it is generally admitted that 10 to 15% of CHM transform into GTTs [27, 28]. This last pattern is particularly interesting to analyze in our study where 30% of all CHM diagnosed at histopathology evolved towards GTT. This is over twofold the numbers reported in the literature and we could argue that late gestational age at diagnosis was a key element of GTT development, as demonstrated by the large majority of GTD initial diagnosis after 12 weeks (73%) among the 11 GTT patients. Several cultural, social, and economic factors could contribute to diagnosis delay in our context, including

- poor early prenatal care: indeed, in addition to the insufficient coverage of public prenatal care services in Morocco, first-trimester routine consultation being not common outside of warning signs for cultural reasons, as local culture encourages “discretion” around early pregnancy, putting women at risk of delayed diagnosis for early pregnancy related complications;
- ignorance of the existence of these complications, associated with the high level of illiteracy among our patients (92% in our series);
- cost constraints related to transportation and user-fees for some healthcare services and tests in public health hospitals. This was the main driver for canceling all fees for GTD patients and ensuring adherence to the treatment and surveillance.

The correlation between delay in diagnosis and negative outcomes for GTD has clearly been established by several authors. In their risk factors' investigations, Karimi-Zarchi et al. demonstrated that the interval between diagnosis and treatment was a key prognosis determinant for the evolution of the disease [18]. Similarly, Lurain et al. showed that 60% of patients dead from GTT had an interval of 4 or more months between diagnosis and treatment, while this interval was found for 20% only of those cured [2].

### 4.3. Creating Innovative Ways to Personalize and Improve Patients' Care

An extremely positive outcome of this program was the customization of patients' care and the subsequent excellent adherence rate (94%) to the full surveillance protocol obtained.

The mean outreach calls or text messages of 2.8 per patient with extremes ranging from 1 to 6 showed that all patients were at risk of dropping out and needed a reminder at least once to fully complete their surveillance protocol. This further supports the crucial need for proactive personalized monitoring of GTD in our context, given the literacy and challenging socioeconomic realities of our patients. Indeed, the studies of Cisse et al., Song et al., and Felemban et al. highlighted this challenge in resource-limited settings by reporting the frequency of negative evolutions and complications due to insufficient monitoring [29–31]. Karimi-Zarchi et al. insisted on quality monitoring for early detection of GTT, key to a better prognosis [18], and Clark et al. emphasized the role played by distance traveled to access care as a key determinant of delayed diagnosis of complications and higher incidence of high risk GTT [32]. These challenges are common in resource-limited settings and various components of our program were instrumental in addressing them. Centralizing paraclinical examinations to allow on-site efficient and rapid management of patients; creating the Electronic Registry to show the list of patients scheduled for control every week and help detect potential delays or missed appointments on a weekly basis; and adopting a proactive approach to patients' management by reaching out to them directly through phone calls or text messages were all pertinent and easily duplicable measures to improve quality of care in GTD management.

### 4.4. Limitations and Challenges

Analysis of this 3-year experience brought to light several challenges faced by our program.

(i) The target of full adherence for all 50 patients was not met, as we missed the goal for 3 of them. The first patient dropped out at an early stage of the surveillance protocol. She lived in another region (*Meknes-Tafilalet*, located approximately 200 miles away from the city of Rabat), which was probably the cause of nonadherence, due to the costs incurred by regular traveling to Rabat to complete her surveillance. Creating follow-up mechanisms in other healthcare structures outside the region of* Rabat-Salé-Zemmour-Zaërs* for the patients receiving their initial management in our Center is an essential way forward for the program to grow and consistently expand. This could be done through implementing similar programs, following the same steps and procedures in other regions, particularly those where patients tend to come to Rabat to seek quality healthcare services.

We also reported 2 cases of contraceptive failure. Both were patients under 25, recently married, and nulliparous. They had voluntarily stopped their contraception to become pregnant again. This raised the issue of the standardized content of our IEC component and the need to further adapt the messaging to patients' specific realities and needs. Di Mattei et al. explored in their recent work the representations and perceptions of disease among GTD and GTT patients [33]. Several models highlight how these perceptions and beliefs become a key element of psychological adaptation of patients to their condition and adherence to the therapeutic project [34, 35]. Their study found a significant impact on patients' quality of life; a major stress related to their fertility prognosis, particularly among nulliparous patients; and a clear tendency to depression and anxiety [33]. This could explain the attitude of rushing into a new pregnancy for our 2 patients, especially in a cultural context where proof of fertility is essential for every young married woman. The key lesson here was the need to integrate a psychological care component into our program, using standardized and validated tools to appreciate patients' perception of their experience, quality of life, and defense mechanisms developed, in order to ensure a better quality and more comprehensive and patient-specific care.

(ii) The delay in GTD diagnosis stood also as a consistent obstacle in optimal GTD care, leading to easily preventable complications. While its complex and intricate social, cultural, and economic drivers would be difficult to address at the level of our Center, awareness-raising campaigns and educational sessions encouraging women to seek prenatal care early during pregnancy could be developed as part of the overall IEC curricula of the Center, targeting the patients attending the different departments (Family Planning, Pregnancies with High Risk, etc.)

(iii) Lastly, the absence of referent histopathologist in GTD was a true limitation to the development of the program. This led us at the design stage to adopt a unified one-year surveillance scheme for all patients, regardless of the histopathological examination outcome. While recognizing the benefit of this measure in ensuring enhanced security for our patients, it is important to highlight the crucial need for in-depth GTD-specific training for histopathologists in order to avoid unnecessary follow-up and expenses in laboratory tests and achieve better cost-effectiveness and possibility of program duplication in other resource-limited settings [36].

### 4.5. Way Forward

In the short run, working on the challenges identified through this evaluation is our first goal, in order to further strengthen the program in the Center.

We would primarily focus on the specific training needs and IEC components highlighted in our analysis. This would in the long-term open the possibility of duplicating the program in other healthcare structures, at the regional and national levels, by organizing targeted training sessions introducing the program, its organization, management, and materials.

Our acquired expertise through this 3-year experience would support the creation of a network of GTD reference centers and would raise our Center to the level of National Observatory, centralizing nation-wide data, sharing skills and expertise at the national level, and providing periodic quality training for practitioners involved in GTD management.

## 5. Conclusion

GTD management requires a competent healthcare structure, with clear diagnosis, treatment, and surveillance guidelines, to ensure an optimal care for patients. Our study documented the first experience of implementing a GTD management program in a public healthcare setting in Morocco. This experience responds to the advocacy calls of various GTD management Centers of Excellence for the creation of reference healthcare structures, with a well-established organization, a codified and comprehensive management program, and proactive surveillance mechanisms, based on a customized model of patient-practitioner partnership. Our program is a simple, cost-effective, and easily duplicable model for settings with similar characteristics and constraints and has the potential to greatly contribute to organizing and improving GTD management in such settings.

## Figures and Tables

**Table 1:** Patients' epidemiological data.

*Age groups (years)*	*<25*	*25–34*	*35–44*	*>45*
	*32%*	26%	14%	28%

*Geographic origin*	*Rabat-Salé-Zemmour-Zaërs region*			*Others* ^*∗*^
	*Rabat*	*Salé*	*Témara*	16%
	12%	*66%*	6%	

*Socioeconomic characteristics*	*Low socioeconomic status*		*Illiteracy*	
	82%		92%	

*GTD management*				

*Gestational age at diagnosis*	*[5–11 weeks + 6 days]*		*[12–24 weeks]*	
	66%		*34%*	

*Histopathology outcome*	*CHM*		*PHM*	
	*68%*		32%	

*Patients' evolution*	*Cured*		*GTT*	
	76%		22%	

^*∗*^ *Three neighboring regions*.

**Table 2:** Characteristics of the 11 GTT patients.

*(i) Gestational age at initial GTD diagnosis*	*<11 weeks + 6 days*	*>12 weeks*
	27%	73%

*(ii) Histopathological outcome*	*CHM*	*PHM*
	*100%*	0%

*(iii) FIGO staging *	*Low risk*	*High risk*
	*91%*	9%
